# Insecticide resistance in Australian *Spodoptera frugiperda* (J.E. Smith) and development of testing procedures for resistance surveillance

**DOI:** 10.1371/journal.pone.0263677

**Published:** 2022-02-10

**Authors:** Lisa Bird, Melina Miles, Adam Quade, Helen Spafford

**Affiliations:** 1 NSW Department of Primary Industries, Tamworth Agricultural Institute, Calala, New South Wales, Australia; 2 Queensland Department of Agriculture and Fisheries, Toowoomba, Queensland, Australia; 3 Department of Primary Industries and Regional Development, Frank Wise Institute of Tropical Agriculture, Kununurra, Western Australia, Australia; Institut Sophia Agrobiotech, FRANCE

## Abstract

*Spodoptera frugiperda* (J.E. Smith) is a highly invasive noctuid pest first reported in northern Australia during early 2020. To document current status of resistance in *S*. *frugiperda* in Australia, insecticide toxicity was tested in field populations collected during the first year of establishment, between March 2020 and March 2021. Dose-response was measured by larval bioassay in 11 populations of *S*. *frugiperda* and a susceptible laboratory strain of *Helicoverpa armigera*. Emamectin benzoate was the most efficacious insecticide (LC_50_ 0.023μg/ml) followed by chlorantraniliprole (LC_50_ 0.055μg/ml), spinetoram (LC_50_ 0.098μg/ml), spinosad (LC_50_ 0.526μg/ml), and methoxyfenozide (1.413μg/ml). Indoxacarb was the least toxic selective insecticide on *S*. *frugiperda* (LC_50_ 3.789μg/ml). Emamectin benzoate, chlorantraniliprole and methoxyfenozide were 2- to 7-fold less toxic on *S*. *frugiperda* compared with *H*. *armigera* while spinosyns were equally toxic on both species. Indoxacarb was 28-fold less toxic on *S*. *frugiperda* compared with *H*. *armigera*. There was decreased sensitivity to Group 1 insecticides and synthetic pyrethroids in *S*. *frugiperda* compared with *H*. *armigera*: toxicity was reduced up to 11-fold for methomyl, 56 to 199-fold for cyhalothrin, and 44 to 132-fold for alpha cypermethrin. Synergism bioassays with metabolic inhibitors suggest involvement of mixed function oxidase in pyrethroid resistance. Recommended diagnostic doses for emamectin benzoate, chlorantraniliprole, spinetoram, spinosad, methoxyfenozide and indoxacarb are 0.19, 1.0, 0.75, 6, 12 and 48μg/μl, respectively.

## Introduction

*Spodoptera frugiperda* (J.E. Smith) (Lepidoptera: Noctuidae) is endemic to tropical and subtropical regions of the Americas [1,2]. However, this species is also highly invasive and has now become a truly global pest having expanded its geographical range to Africa [3], Asia [4–7] and Australia where it was first reported in 2020 [8]. It rapidly established across tropical and subtropical regions of Australia including north Queensland, Northern Territory, and northern parts of Western Australia. Predictions suggest that *S*. *frugiperda* populations are likely to persist year-round throughout cropping regions of northern Australia with seasonal migrations from this permanent range likely to extend into more southerly agricultural regions [9] where they are likely to produce multiple and overlapping generations per year [10,11] across a broad host range throughout vegetative and reproductive stages of crop development [12]. A further challenge is that larvae become concealed in protected feeding sites such as the whorls and ears of plants which makes chemical control difficult to achieve.

The pest status of *S*. *frugiperda* is enhanced by a capacity to develop insecticide resistance. Reliance on chemical control strategies on a global scale over many decades has led to the development of resistance to at least 29 insecticidal active ingredients in six mode of action groups [13,14]. Field-evolved resistance to proteins from the bacterium *Bacillus thuringiensis* (Bt) has also been documented in populations of *S*. *frugiperda* from the Americas [15]. The widespread development of Bt-resistant populations of *S*. *frugiperda* has the potential to place further selection pressure on synthetic insecticides used in the management of this pest across a range of cropping systems, particularly insecticides which have a high compatibility with integrated pest management (IPM). Currently in Australia, growers are reliant on chemical intervention to suppress populations of *S*. *frugiperda* because of limited non-chemical options for management. However, the extensive use of chemical control can have detrimental impacts on non-target organisms [16] and even insecticides considered to have high pest specificity can also have lethal and sublethal effects on beneficial arthropods [17–19]. Nevertheless, insecticide use is currently the mainstay for management of *S*. *frugiperda* globally and judicious use of a range of selective chemical options and non-chemical control measures within an IPM framework will be the most effective strategy for management of *S*. *frugiperda* [20,21].

The reported mechanisms of insecticide resistance in *S*. *frugiperda* include target site insensitivity where a small number of highly conserved point mutations in genes encode for receptor target sites such the ryanodine receptor (RyR) which confers resistance to diamide insecticides [22], acetylcholinesterase (AChE) which confers resistance to carbamates and organophosphates, and voltage-gated sodium channel (VGSC) which confers resistance to synthetic pyrethroids [22,23]. Metabolic detoxification has also been implicated in resistance to pyrethroids, organophosphates and carbamates. This mechanism involves genomic changes which cause gene amplification, overexpression and/or modification of genes encoding glutathione S-transferases, cytochrome P450s and carboxylesterases [22–25].

The situation is confounded in Australia because of the pre-existing pest complex, particularly *Helicoverpa armigera*, which is a long-standing target for management by growers in several commodities such as cotton and grains [26–29] and is already at high risk of developing resistance to insecticides [30]. There is considerable potential for multi-species selection for resistance in the field if there is an increase in frequency of insecticide applications in broadacre crops where *S*. *frugiperda* and *H*. *armigera* occur together such as in maize and sorghum, and in areas where these and other host crops are grown in rotational systems.

To maintain the security of agricultural production in Australia it will be crucial to monitor susceptibility of *S*. *frugiperda* to available control strategies including consideration of ecological factors such as cultivated and uncultivated host preference and the impact of management practices on selection for resistance in these preferred hosts. Movement of populations out of treated environments as a result of migratory events within Australia as well as incursions from neighbouring countries, will be important for determining the rate at which resistance genes will spread [31,32].

As a first step in resistance management of *S*. *frugiperda* in Australia, particularly to insecticidal groups that are highly effective and pivotal to IPM in this pest, it was necessary to determine inherent susceptibility as a baseline for measuring future changes in sensitivity to insecticides. An understanding of the criteria for resistance also provides the opportunity to monitor the long-term effectiveness of resistance management strategies. Therefore, the objectives of this study were firstly to evaluate baseline response in field collected populations of *S*. *frugiperda* to insecticides currently registered for control of this pest in Australia and to use the data generated from these bioassays to establish diagnostic concentrations of insecticides. Secondly, to understand the practical significance of the baseline response, we used a laboratory susceptible strain of *H*. *armigera* as a standard for comparing insecticide efficacy in Australian *S*. *frugiperda*. Thirdly, we investigated the synergistic effects of enzyme inhibitors to determine whether metabolic detoxification plays a role in pyrethroid resistance in Australian populations of *S*. *frugiperda*. The implications of these results for management of *S*. *frugiperda* in Australia are discussed.

## Materials and methods

### Ethics statement

All insect samples were collected from agricultural land cultivated for the purpose of commercial maize production and hence no specific permits were required to conduct sampling in any of the locations referred to in this study. The species studied is an agricultural pest with world-wide distribution and hence is not classified as an endangered or protected species.

### Insect strains

Populations of *S*. *frugiperda* were sampled during the first year of its establishment in Australia from March 2020 to March 2021 and were used to establish baseline response to insecticides registered for use on this species in Australia. Larvae were collected from maize crops in northern, central and southern cropping regions of Queensland as well as Kununurra in northern Western Australia (Table 1) to produce 11 strains for bioassay. A minimum of 30 larvae constituted any one geographically distinct strain and populations were tested within four generations of establishment in the laboratory.

**Table 1:** Populations of *Spodoptera frugiperda* used to characterise the response to insecticides.

Strain	Region	GPS Location	Collection Date	Host
Walkamin	Atherton Tablelands	-17°8’13", 145°25’44"	25.3.20	Maize
Mackay	Mackay	-21°18’21", 148°59’46"	27.7.20	Maize
Bowen	Bowen	-20°02’50", 148°07’12"	14.10.20	Sweet corn
Ayr	Burdekin	-19°37’05", 147°22’30"	31.11.20	Maize
Chinchilla	Darling Downs	-26°49’56", 150°24’21"	29.10.20	Maize
Dalby	Darling Downs	-27°13’59", 151°20’19"	14.1.21	Maize
Wheatvale	Darling Downs	-28°08’60", 151°51’43"	27.1.21	Maize
Nandi	Darling Downs	-27°15’23", 151°07’25"	5.2.21	Maize
Mount Tarampa	Lockyer Valley	-27°27’52", 152°30’41"	12.2.21	Sweet corn
Byee	Bundaberg	-26°11’36", 151°50’44"	31.3.21	Maize
Kununurra	Ord River	-15°43’58", 128°44’49"	1.4.20–29.1.21	Maize, sweet corn, sorghum

The response of *S*. *frugiperda* was compared with bioassay results from a laboratory strain of *H*. *armigera* known as the New GR strain which has never been exposed to insecticides. This strain was established in 2011 from a cohort of a general laboratory (GR strain) of *H*. *armigera*. The progenitor GR strain was sourced during the mid-1980s from a series of collections from cotton fields in the Namoi Valley, northern New South Wales, Australia.

### Insect rearing

Field collected larvae of *S*. *frugiperda* and larvae from the *H*. *armigera* laboratory strain were reared on artificial diet adapted from Akhurst et al. (2003) [33]. Larvae were maintained in rearing trays (Tacca Plastics, Sydney, Australia) which were covered and heat-sealed with perforated lids (Oliver Products, Grand Rapids, MI, USA). Moths of *S*. *frugiperda* and *H*. *armigera* were provided a 4% honey/sugar solution fed through a cotton wick. Moths were housed in containers open at the top and covered with cloth liners secured around the lip which provided an oviposition substrate; eggs were harvested every two to three days.

Eggs of *H*. *armigera* were removed from cloth liners by washing in 1% bleach solution and collected onto filter papers by vacuum filtration. Filter papers were air dried and placed in sealed plastic bags until neonates hatched. Neonate larvae of *H*. *armigera* were transferred individually to rearing trays to prevent losses due to cannibalism. Egg masses of *S*. *frugiperda* were cut from the cloth liners and suspended above artificial diet in 250ml round containers. Larvae of *S*. *frugiperda* were reared to the third or fourth instar before being transferred individually into rearing trays. Insect strains were maintained throughout their lifecycle at 26 ± 1°C with 14:10 (L: D) h photoperiod and ambient RH.

### Insecticides

The insecticide mode of action groups tested in this study were registered under permit for *S*. *frugiperda* control (Table 2). Selective groups were supplied as formulated insecticides: emamectin benzoate (Affirm [1.9% active ingredient]) was provided by Syngenta Crop Protection; chlorantraniliprole (Altacor [35% active ingredient) and indoxacarb (Steward [15% active ingredient]) were provided by DuPont Australia Ltd., Macquarie Park, Australia; spinetoram (Success Neo [12% active ingredient]) and spinosad (Entrust [24% active ingredient]) were provided by Corteva Agriscience, Chatswood, Australia. Broad-spectrum mode of action groups were supplied as technical grade insecticide: alpha-cypermethrin (99.5%) and gamma-cyhalothrin (99.9%) were provided by FMC, North Ryde, Australia; methomyl (98.0%), chlorpyrifos (98.0%) and piperonyl butoxide (PBO) (90%) triphenyl phosphate (TPP) (99%) and diethyl maleate (DEM) (96%) were supplied by Sigma-Aldrich Pty Ltd., North Ryde, Australia.

**Table 2:** Summary of permits for *Spodoptera frugiperda* control as of July 2021.

Active Constituent	MOA Group	Permit number
Methomyl	1A	PER89279, PER89293, PER89400, PER89330
Alpha-cypermethrin	3A	PER89279, PER85447, PER89425, PER89330
Gamma-cyhalothrin	3A	PER89358
Spinetoram	5	PER89241, PER89331, PER89327, PER89284, PER89390, PER90737
Spinosad	5	PER89870
Emamectin benzoate	6	PER89285, PER89263, PER89300, PER89344, PER89371, PER89330
Indoxacarb	22A	PER89306, PER89279, PER89278, PER89311, PER89530, PER89286, PER89705, PER89330, PER90577, PER90374
Chlorantraniliprole	28	PER86014, PER89290, PER89366, PER89281, PER89353, PER89384, PER89259, PER89354, PER89457, PER89330, PER90758, PER90621, PER89280

### Insect bioassays

The selective insecticides (indoxacarb, chlorantraniliprole, emamectin benzoate, spinetoram, spinosad and methoxyfenozide) are known to be more toxic in lepidopteran larvae by ingestion than by contact [34–37]). Therefore, bioassays were performed by exposing larvae to artificial diet into which formulated insecticide was incorporated. Formulated insecticides were used to produce two-fold serial dilutions in distilled water. The dose range included six or seven insecticide concentrations were that were expected to induce from 1 to 100% mortality. The insecticide solutions were added to 200ml of diet and a homogenous mixture of diet and insecticide was created by vigorous shaking for 30 seconds. Diet was dispensed into 45-well bioassay trays (Tacca Plastics, Moorebank, Australia); individual wells contained approx. 1ml of diet. Larvae of *S*. *frugiperda* and *H*. *armigera* were reared on untreated diet to the late second or third instar then introduced to trays with treated diet, one larva per well.

The dose response to broad-spectrum contact insecticides (synthetic pyrethroids, organophosphate and carbamate) was tested by topical application of insecticide as a backline treatment to *S*. *frugiperda* and *H*. *armigera* larvae. Test solutions were prepared from technical grade insecticide dissolved in acetone to produce six to eight two-fold serial dilutions expected to induce 0–100% mortality. Larvae of both species were reared on untreated artificial diet and third or fourth instar larvae within a weight range of 30-40mg were selected for testing. Bioassays were performed by topical administration of 1μl of acetone/insecticide solution applied to the dorsal thorax of larvae using a 50μl micro-syringe in a repeating dispenser (Hamilton Company, Reno, NV, USA).

Synergism bioassays were performed according to the method of Forrester et al. (1993). Test solutions of PBO, TPP and DEM were prepared by dissolving analytical grade reagent in acetone to produce a concentration known to cause no mortality in *S*. *frugiperda* or *H*. *armigera* (50μg/μl for each synergist). Larvae were then treated topically with 1μl of acetone/synergist solution, as above, followed by topical application of 1μl of insecticide.

Bioassay trays containing test insects were covered with heat-sealed perforated lids (Unipac Solutions, Albion Park, NSW, Australia). All bioassays were replicated a minimum of three times with each replicate consisting of 20 individuals. Acetone alone was used as the control in topical bioassays, synergists dissolved in acetone at concentration of 50μg/μl were used as the control in inhibition bioassays, and untreated diet was used as the control in diet incorporation bioassays. Bioassays were maintained for seven days under the same conditions described above for larval rearing and assessed for mortality using one or more of the following criteria: larvae unable to demonstrate coordinated movement when prodded with a blunt probe; paralysis of prolegs; larvae very slow to right themselves (time exceeding three seconds).

### Data analysis

Bioassay data were analysed using a stand-alone probit program [38] applying source codes that were developed by implementing previously described methods [39] including data adjustment for control mortality [40], to estimate LC_50_, LC_99.9_, associated slope values and 95% fiducial limits (FLs). Significant differences (P = 0.05) between LC_50_ values were determined by the lethal concentration ratio test where if the 95% confidence interval (CI) includes 1 then the LC_50_s were not significantly different [41]. The toxicity ratio of insecticides was calculated by dividing the LC_50_ of each insecticide in each *S*. *frugiperda* population by the LC_50_ of each insecticide in the laboratory strain of *H*. *armigera*. Synergism ratio (SR) was calculated by dividing the LC_50_ of the strain tested with pyrethroid alone by LC_50_ of the strain tested with pyrethroid and synergist treatment.

## Results

### Variability in insecticide toxicity between *S*. *frugiperda* populations

Emamectin benzoate was the most toxic insecticide with an average LC_50_ of 0.023μg/ml. A narrow (1.7-fold) range of variability was associated with the dose response to emamectin benzoate in 11 geographically diverse populations of *S*. *frugiperda*, ranging from 0.017 μg/ml (Dalby and Nandi strains) to 0.029 μg/ml (Kununurra strain) (Table 3). Chlorantraniliprole was also highly toxic to *S*. *frugiperda* with an average LC_50_ of 0.055μg/ml which ranged from 0.039μg/ml (Byee strain) to 0.091μg/ml (Ayr strain), representing low (2.3-fold) variability between populations (Table 3).

**Table 3:** Bioassay of field strains of *S*. *frugiperda* and one laboratory susceptible strain of *H*. *armigera* tested as late second/early third instars on insecticide-incorporated artificial diet containing indoxacarb, chlorantraniliprole and emamectin benzoate and assessed for mortality at 7 days.

Insecticide	Species	Strain	*n*	LC_50_ [mg ai L^-1^] (95% FL)	LC_99.9_ [mg ai L^-1^]	Toxicity ratio†	Fit of probit line			% Mortality at proposed diagnostic concentrations [mg ai L^-1^] (*n*)		
							Slope ± SE	*Χ*^2^ (df)	*P*			
Emamectin benzoate										0.0475	0.095	0.19^‡^
	*S*. *frugiperda*	Walkamin	299	0.018 (0.016, 0.020)	0.11	2	3.9 ± 0.3	4.85 (4)	0.303	94.7 (150)	100 (150)	100 (150)
	*S*. *frugiperda*	Mackay	357	0.023 (0.020, 0.026)	0.18	2	3.4 ± 0.3	5.68 (3)	0.128	82.8 (192)	95.8 (192)	100 (194)
	*S*. *frugiperda*	Bowen	360	0.027 (0.017, 0.044)	0.30	3	3.0 ± 0.5	9.34 (3)	0.025	76.7 (150)	98.7 (149)	100 (150)
	*S*. *frugiperda*	Ayr	417	0.028 (0.025, 0.032)	0.23	3	3.4 ± 0.3	6.71(4)	0.152	79.7 (148)	95.3 (150)	100 (150)
	*S*. *frugiperda*	Chinchilla	360	0.019 (0.017, 0.021)	0.09	2	4.5 ± 0.5	0.14 (3)	0.987	96.7 (60)	100 (60)	100 (60)
	*S*. *frugiperda*	Dalby	259	0.017 (0.015, 0.020)	0.14	2	3.4 ± 0.4	3.26 (2)	0.196	83.5 (194)	94.9 (195)	100 (194)
	*S*. *frugiperda*	Nandi	334	0.017 (0.010, 0.028)	0.21	2	2.8 ± 0.5	10.10 (3)	0.018	83.3 (150)	98.6 (147)	100 (186)
	*S*. *frugiperda*	Mount Tarampa	379	0.024 (0.022, 0.028)	0.20	3	3.4 ± 0.3	0.36 (4)	0.986	81.7 (60)	98.3 (60)	100 (60)
	*S*. *frugiperda*	Wheatvale	260	0.025 (0.021, 0.028)	0.17	3	3.7 ± 0.4	0.03 (2)	0.985	72.8 (195)	96.4 (195)	100 (194)
	*S*. *frugiperda*	Byee	258	0.026 (0.023, 0.029)	0.16	3	3.9 ± 0.4	4.59 (2)	0.101	85.3 (150)	99.3 (148)	100 (147)
	*S*. *frugiperda*	Kununurra	316	0.029 (0.025, 0.033)	0.26	3	3.2 ± 0.3	3.49 (3)	0.322	72.7 (194)	97.9 (195)	100 (194)
	*S*. *frugiperda*	Pooled	3599	0.023 (0.019, 0.026)	0.22	3	3.1 ± 0.2	26.08 (4)	<0.01	81.5 (1643)	97.4 (1641)	100 (1679)
	*H*. *armigera*	New GR	335	0.009 (0.006, 0.012)	0.04	-	4.8 ± 0.9	8.25 (3)	0.041	100 (259)	100 (219)	-
Chlorantraniliprole										0.25	0.5	1.0^‡^
	*S*. *frugiperda*	Walkamin	418	0.080 (0.070, 0.093)	0.95	4	2.9 ± 0.2	5.32 (4)	0.256	94.0 (149)	100 (149)	100 (89)
	*S*. *frugiperda*	Mackay	411	0.079 (0.068, 0.093)	1.38	4	2.5 ± 0.2	4.72 (4)	0.317	94.1 (186)	99.5 (192)	100 (239)
	*S*. *frugiperda*	Bowen	413	0.071 (0.062, 0.082)	0.87	4	2.8 ± 0.2	1.62 (4)	0.805	94.9 (195)	99.5 (192)	100 (195)
	*S*. *frugiperda*	Chinchilla	420	0.051 (0.042, 0.060)	1.24	3	2.2 ± 0.2	1.69 (4)	0.792	91.7 (60)	98.3 (60)	100 (60)
	*S*. *frugiperda*	Ayr	356	0.091 (0.079, 0.104)	0.78	5	3.3 ± 0.3	2.45 (3)	0.484	96.6 (206)	100 (208)	100 (206)
	*S*. *frugiperda*	Dalby	255	0.044 (0.039, 0.050)	0.33	2	3.5 ± 0.4	0.37 (2)	0.831	97.9 (238)	99.2 (237)	100 (238)
	*S*. *frugiperda*	Nandi	338	0.039 (0.033, 0.045)	0.42	2	3.0 ± 0.3	1.69 (3)	0.639	99.3 (149)	100 (149)	100 (150)
	*S*. *frugiperda*	Mount Tarampa	480	0.075 (0.063, 0.088)	1.63	4	2.3 ± 0.2	4.73 (5)	0.450	88.3 (60)	96.7 (60)	100 (60)
	*S*. *frugiperda*	Wheatvale	257	0.040 (0.034, 0.046)	0.41	2	3.1 ± 0.3	2.56 (2)	0.278	96.7 (180)	100 (191)	100 (191)
	*S*. *frugiperda*	Byee	282	0.039 (0.035, 0.044)	0.19	2	4.4 ± 0.5	5.86 (2)	0.053	99.5 (194)	100 (195)	100 (193)
	*S*. *frugiperda*	Kununurra	314	0.047 (0.041, 0.055)	0.60	3	2.8 ± 0.3	6.86 (3)	0.076	99.5 (193)	100 (195)	100 (195)
	*S*. *frugiperda*	Pooled	3944	0.055 (0.052, 0.058)	1.04	3	2.4 ± 0.1	4.36 (2)	0.113	96.5 (1810)	99.6 (1828)	100 (1816)
	*H*. *armigera*	New GR	299	0.018 (0.015, 0.020)	0.12	-	3.7 ± 0.4	4.24 (2)	0.374	100 (288)	-	-
Indoxacarb										12.0^‡^	24.0	48.0
	*S*. *frugiperda*	Walkamin	539	1.480 (1.216, 1.778)	64.30	11	1.9 ± 0.2	2.08 (6)	0.912	95.8 (237)	99.2 (240)	100 (239)
	*S*. *frugiperda*	Mackay	679	4.499 (3.976, 5.094)	91.17	33	2.4 ± 0.2	7.00 (4)	0.136	84.0 (263)	96.5 (398)	100 (392)
	*S*. *frugiperda*	Bowen	360	5.387 (4.514, 6.403)	133.47	40	2.2 ± 0.2	7.40 (3)	0.060	83.8 (253)	98.4 (255)	100 (255)
	*S*. *frugiperda*	Ayr	359	8.408 (7.162, 9.788)	119.88	63	2.7 ± 0.3	6.41 (3)	0.093	78.0 (209)	92.3 (208)	100 (209)
	*S*. *frugiperda*	Chinchilla	359	3.220 (2.753, 3.773)	52.00	24	2.6 ± 0.2	1.67 (3)	0.644	95.0 (60)	98.3 (60)	100 (60)
	*S*. *frugiperda*	Dalby	320	3.127 (2.585, 3.695)	58.80	23	2.4 ± 0.3	4.94 (3)	0.176	86.6 (239)	97.9 (239)	100 (239)
	*S*. *frugiperda*	Nandi	299	5.699 (4.874, 6.564)	52.59	43	3.2 ± 0.4	0.40 (2)	0.980	99.3 (150)	99.3 (150)	100 (150)
	*S*. *frugiperda*	Mount Tarampa	320	3.578 (3.040, 4.160)	47.98	27	2.7 ± 0.3	3.42 (3)	0.331	90.0 (60)	100 (60)	100 (60)
	*S*. *frugiperda*	Wheatvale	379	2.310 (1.877, 2.787)	96.02	17	1.9 ± 0.2	1.77 (4)	0.778	90.7 (195)	96.0 (149)	100 (148)
	*S*. *frugiperda*	Byee	319	2.557 (2.134, 3.044)	66.09	19	2.2 ± 0.2	4.99 (3)	0.172	98.7 (150)	99.3 (194)	100 (193)
	*S*. *frugiperda*	Kununurra	359	8.141 (7.028, 9.374)	85.05	61	3.0 ± 0.3	4.41 (3)	0.220	79.5 (195)	96.9 (194)	100 (195)
	*S*. *frugiperda*	Pooled	4292	3.789 (3.588, 3.994)	99.66	28	2.2 ± 0.1	2.35 (3)	0.503	88.2 (2011)	97.4 (2148)	100 (2142)
	*H*. *armigera*	New GR	300	0.134 (0.119, 0.152)	0.80	-	4.0 ± 0.4	5.92 (2)	0.052	100 (180)	-	-

† LC_50_ *S*. *frugiperda*/LC_50_ *H*. *armigera*.

‡ Indicates diagnostic concentration in *H*. *armigera*.

Indoxacarb was significantly less toxic than emamectin benzoate and chlorantraniliprole with an average LC_50_ of 3.789μg/ml. There was also higher variability (5.7-fold) in dose response to indoxacarb between the 11 populations of *S*. *frugiperda* tested, with the most sensitive strain from Walkamin (1.480μg/ml) and the most tolerant strain from Ayr (8.408 μg/ml) (Table 3).

Larvae of *S*. *frugiperda* were highly susceptible to spinetoram (average LC_50_ of 0.098μg/ml) and the response was highly consistent among populations of *S*. *frugiperda* with only a 1.6-fold difference between the most sensitive and most tolerant strains (Table 4). There was also a narrow range of variability (2.4-fold) in spinosad toxicity between strains; the Kununurra strain was the most sensitive and the Wheatvale strain was the most tolerant to both spinosyn insecticides (Table 4). However, spinosad was 5.4-fold less toxic to *S*. *frugiperda* (average LC_50_ 0.526μg/ml) than spinetoram. Methoxyfenozide had an average LC_50_ of 1.413μg/ml and a similar narrow range of variation (3.3-fold) with LC_50_ values ranging from 0.805 μg/ml (Nandi strain) to 2.634 μg/ml (Bowen strain) (Table 4).

**Table 4:** Bioassay of field strains of *S*. *frugiperda* and one laboratory susceptible strain of *H*. *armigera* tested as late second/early third instars on insecticide-incorporated artificial diet containing spinetoram, spinosad and methoxyfenozide and assessed for mortality at 7 days.

Insecticide	Species	Strain	*n*	LC_50_ [mg ai L^-1^] (95% FL)	LC_99.9_ [mg ai L^-1^]	Toxicity ratio†	Fit of probit line			% Mortality at candidate diagnostic concentrations [mg ai L^-1^] (*n*)		
							Slope ± SE	*Χ*^2^ (df)	*P*			
Spinetoram										0.1875	0.375	0.75¥
	*S*. *frugiperda*	Walkamin	240	0.088 (0.081, 0.096)	0.21	1	8.1 ± 1.3	0.39 (1)	0.532	97.4 (195)	100 (194)	100 (195)
	*S*. *frugiperda*	Mackay	299	0.116 (0.104, 0.129)	0.45	1	5.2 ± 0.6	0.48 (2)	0.787	87.9 (149)	99.3 (149)	100 (150)
	*S*. *frugiperda*	Bowen	300	0.110 (0.098, 0.123)	0.48	1	4.8 ± 0.5	3.70 (2)	0.157	79.9 (194)	98.5 (195)	100 (193)
	*S*. *frugiperda*	Ayr	300	0.082 (0.074, 0.090)	0.26	1	6.1 ± 0.7	0.06 (2)	0.970	92.1 (240)	100 (239)	100 (240)
	*S*. *frugiperda*	Dalby	240	0.090 (0.082, 0.099)	0.29	1	6.1 ± 0.7	0.47 (1)	0.493	97.1 (104)	100 (105)	100 (105)
	*S*. *frugiperda*	Mount Tarampa	320	0.085 (0.074, 0.096)	0.61	1	3.6 ± 0.3	1.53 (3)	0.675	90.0 (60)	100 (60)	100 (60)
	*S*. *frugiperda*	Nandi	277	0.105 (0.092, 0.119)	0.64	1	3.9 ± 0.4	3.45 (2)	0.178	93.8 (240)	99.6 (237)	100 (285)
	*S*. *frugiperda*	Wheatvale	319	0.127 (0.109, 0.148)	1.66	1	2.8 ± 0.3	4.26 (3)	0.235	70.5 (105)	95.2 (105)	100 (105)
	*S*. *frugiperda*	Byee	259	0.106 (0.093, 0.121)	0.75	1	3.6 ± 0.4	1.14 (2)	0.565	87.1 (194)	99.0 (194)	100 (192)
	*S*. *frugiperda*	Kununurra	260	0.080 (0.072, 0.090)	0.31	1	5.2 ± 0.6	2.69 (2)	0.260	96.0 (150)	99.3 (150)	100 (147)
	*S*. *frugiperda*	Pooled	2733	0.098 (0.095, 0.102)	0.54	1	4.2 ± 0.1	3.36 (2)	0.186	89.8 (1631)	99.2 (1628)	100 (1672)
	*H*. *armigera*	New GR	416	0.088 (0.077, 0.100)	0.71	-	3.4 ± 0.3	2.77 (4)	0.597	90.0 (60)	96.7 (60)	100 (60)
Spinosad										1.5	3.0	6.0¥
	*S*. *frugiperda*	Walkamin	260	0.467 (0.417, 0.526)	2.35	1	4.4 ± 0.5	5.18 (2)	0.075	100 (150)	100 (150)	100 (150)
	*S*. *frugiperda*	Mackay	360	0.435 (0.270, 0.660)	3.27	1	3.5 ± 0.6	9.11 (3)	0.028	95.9 (148)	100 (150)	100 (150)
	*S*. *frugiperda*	Bowen	344	0.438 (0.388, 0.494)	2.37	1	4.2 ± 0.4	3.73 (3)	0.338	94.0 (150)	99.3 (150)	100 (150)
	*S*. *frugiperda*	Ayr	420	0.437 (0.379, 0.504)	5.10	1	2.9 ± 0.2	4.77 (4)	0.312	95.3 (150)	100 (150)	100 (149)
	*S*. *frugiperda*	Dalby	320	0.544 (0.477, 0.618)	4.04	1	3.6 ± 0.3	0.92 (3)	0.821	96.2 (105)	100 (105)	100 (105)
	*S*. *frugiperda*	Nandi	340	0.528 (0.315, 0.864)	3.28	1	3.9 ± 0.8	11.92 (3)	0.008	92.0 (150)	99.3 (150)	100 (150)
	*S*. *frugiperda*	Wheatvale	258	0.998 (0.881, 1.130)	6.03	2	4.0 ± 0.4	0.64 (2)	0.726	71.7 (237)	97.9 (240)	100 (240)
	*S*. *frugiperda*	Byee	260	0.708 (0.627, 0.796)	3.61	1	4.4 ± 0.5	1.14 (2)	0.565	84.7 (150)	98.7 (149)	100 (147)
	*S*. *frugiperda*	Kununurra	259	0.411 (0.362, 0.464)	2.43	1	4.0 ± 0.4	3.83 (2)	0.147	97.3 (150)	100 (150)	100 (150)
	*S*. *frugiperda*	Pooled	3000	0.526 (0.463, 0.596)	4.26	1	3.4 ± 0.2	9.27 (3)	0.026	90.5 (1390)	99.4 (1394)	100 (1391)
	*H*. *armigera*	New GR	420	0.552 (0.478, 0.634)	5.61	-	3.1 ± 0.3	6.37 (4)	0.173	90.0 (60)	96.7 (60)	100 (60)
Methoxyfenozide										3.0	6.0	12.0¥
	*S*. *frugiperda*	Walkamin	538	2.004 (1.232, 3.201)	18.35	5	3.2 ± 0.4	6.53 (2)	0.038	68.6 (118)	96.7 (120)	100 (60)
	*S*. *frugiperda*	Mackay	356	1.800 (0.566, 2.063)	16.35	5	3.2 ± 0.3	5.60 (3)	0.133	67.8 (60)	100 (60)	100 (60)
	*S*. *frugiperda*	Bowen	239	2.634 (2.308, 2.986)	15.32	7	4.0 ± 0.5	0.33 (1)	0.566	61.7 (60)	91.5 (60)	100 (60)
	*S*. *frugiperda*	Ayr	419	1.899 (1.643, 2.192)	22.81	5	2.9 ± 0.2	4.19 (4)	0.381	71.7 (60)	93.3 (60)	100 (60)
	*S*. *frugiperda*	Dalby	320	1.153 (1.018, 1.306)	7.37	3	3.8 ± 0.4	1.46 (3)	0.691	95.0 (60)	100 (60)	100 (60)
	*S*. *frugiperda*	Nandi	340	0.805 (0.530, 1.236)	9.06	2	2.9 ± 0.4	7.85 (3)	0.049	100 (60)	100 (59)	100 (60)
	*S*. *frugiperda*	Wheatvale	317	1.302 (1.151, 1.473)	7.97	3	3.9 ± 0.4	1.73 (3)	0.630	93.3 (60)	100 (60)	100 (60)
	*S*. *frugiperda*	Byee	260	0.755 (0.655, 0.862)	5.83	2	3.5 ± 0.4	0.87 (2)	0.647	98.3 (60)	100 (58)	100 (60)
	*S*. *frugiperda*	Kununurra	315	1.550 (1.357, 1.769)	12.45	4	3.4 ± 0.3	0.35 (3)	0.950	81.7 (60)	98.3 (60)	100 (60)
	*S*. *frugiperda*	Pooled	3281	1.413 (1.213, 1.643)	18.32	4	2.8 ± 0.2	11.72 (3)	0.008	81.2 (597)	97.7 (596)	100 (480)
	*H*. *armigera*	New GR	300	0.386 (0.167, 0.839)	2.21	-	4.1 ± 1.0	11.15 (2)	0.004	100 (60)	100 (60)	-

† LC_50_ *S*. *frugiperda*/LC_50_ *H*. *armigera*.

¥ Proposed diagnostic concentration for *S*. *frugiperda*.

There was also a narrow range of variability in response to broad-spectrum insecticides in geographically different populations of *S*. *frugiperda*. For Group 1 insecticides there was a 2.1-fold range of variability in response to chlorpyrifos between the most sensitive and least sensitive populations (Dalby and Mackay strains, respectively) and a 4.7-fold range of variability in response to methomyl between the most sensitive and least sensitive populations (Byee and Ayr strains, respectively) (Table 5). For synthetic pyrethroids, there was a 3.0-fold range of variability between the most sensitive population (Bowen) and least sensitive population (Kununurra) to alpha cypermethrin, while there was a 3.6-fold variation between the most sensitive population (Walkamin) and least sensitive population (Ayr) to gamma cyhalothrin (Table 5).

**Table 5:** Toxicity of broad-spectrum contact insecticides on *S*. *frugiperda* compared with a laboratory susceptible strain of *H*. *armigera* determined from topical bioassays and assessed for mortality at 3 days.

Insecticide	Species	Strain	*n*	LD_50_ [μg/μl] (95% FL)	LD_99.9_ [μg/μl]	Toxicity ratio†	Fit of probit line			% Mortality at *H*. *armigera* diagnostic concentration [μg/μl] (*n*)
							Slope ± SE	*Χ*^2^ (df)	*P*	
Methomyl	*S*. *frugiperda*	Walkamin	379	0.945 (0.790, 1.340)	30.23	3	2.0 ± 0.2	1.92 (5)	0.589	56.7 (60)
	*S*. *frugiperda*	Mackay	420	1.102 (0.487, 2.135)	406.82	4	1.2 ± 0.2	10.48 (4)	0.033	63.3 (60)
	*S*. *frugiperda*	Bowen	360	2.600 (2.072, 3.342)	247.84	9	1.6 ± 0.2	4.10 (3)	0.251	31.7 (60)
	*S*. *frugiperda*	Ayr	480	3.153 (2.411, 4.242)	1791.94	11	1.1 ± 0.1	7.24 (5)	0.203	33.3 (60)
	*S*. *frugiperda*	Dalby	260	0.839 (0.674, 1.037)	96.56	3	1.5 ± 0.1	10.35 (5)	0.066	58.3 (60)
	*S*. *frugiperda*	Nandi	438	0.988 (0.781, 1.249)	203.50	3	1.3 ± 0.1	3.94 (5)	0.558	46.7 (60)
	*S*. *frugiperda*	Wheatvale	379	1.026 (0.824, 1.303)	109.03	4	1.5 ± 0.2	9.20 (4)	0.056	35.6 (59)
	*S*. *frugiperda*	Byee	382	0.666 (0.466, 0.882)	262.94	2	1.2 ± 0.1	6.78 (4)	0.140	61.5 (65)
	*S*. *frugiperda*	Kununurra	380	1.683 (1.283, 2.242)	689.29	6	1.2 ± 0.1	7.23 (4)	0.124	41.7 (60)
	*S*. *frugiperda*	Pooled	3478	1.270 (1.168, 1.380)	368.71	4	1.2 ± 0.1	8.29 (6)	0.218	47.8 (544)
	*H*. *armigera*	New GR	380	0.283 (0.247, 0.325)	2.75	-	3.1 ± 0.3	4.42 (3)	0.219	96.7 (60)
Chlorpyrifos	*S*. *frugiperda*	Walkamin	440	13.966 (11.750, 16.622)	423.42	3	2.1 ± 0.2	6.18 (5)	0.289	53.3 (60)
	*S*. *frugiperda*	Mackay	259	18.168 (15.826, 20.832)	170.16	4	3.2 ± 0.3	1.19 (3)	0.755	58.3 (60)
	*S*. *frugiperda*	Bowen	300	13.532 (11.520, 15.859)	189.31	3	2.7 ± 0.3	5.15 (2)	0.075	78.3 (60)
	*S*. *frugiperda*	Ayr	300	17.955 (15.677, 20.707)	151.12	4	3.3 ± 0.4	0.06 (2)	0.970	55.0 (60)
	*S*. *frugiperda*	Dalby	480	8.515 (7.495, 9.611)	126.82	2	3.6 ± 0.2	7.21 (3)	0.066	80.0 (60)
	*S*. *frugiperda*	Nandi	378	15.761 (13.829, 18.034)	237.69	4	2.6 ± 0.2	0.61 (2)	0.737	60.0 (90)
	*S*. *frugiperda*	Wheatvale	260	13.744 (12.026, 15.698)	105.98	3	3.5 ± 0.4	1.18 (2)	0.554	73.3 (60)
	*S*. *frugiperda*	Byee	260	12.834 (11.410, 14.424)	66.125	3	4.3 ± 0.5	0.73 (2)	0.694	81.7 (60)
	*S*. *frugiperda*	Kununurra	259	13.323 (11.248, 15.728)	217.38	3	2.6 ± 0.3	0.05 (2)	0.975	67.8 (60)
	*S*. *frugiperda*	Pooled	2936	13.631 (12.981, 14.308)	189.412	3	2.7 ± 0.1	4.53 (3)	0.210	68.0 (609)
	*H*. *armigera*	New GR	360	4.247 (3.616, 4.967)	69.65	-	2.5 ± 0.1	5.47 (3)	0.140	98.3 (60)
Alpha cypermethrin	*S*. *frugiperda*	Walkamin	886	0.676 (0.569, 0.793)	165.76	48	1.3 ± 0.1	2.45 (5)	0.784	15.0 (100)
	*S*. *frugiperda*	Mackay	400	0.821 (0.698, 0.967)	16.77	59	2.4 ± 0.2	3.37 (4)	0.498	0 (40)
	*S*. *frugiperda*	Bowen	380	0.620 (0.529, 0.726)	11.72	44	2.4 ± 0.2	2.34 (4)	0.673	6.7 (60)
	*S*. *frugiperda*	Ayr	360	1.768, 1.025, 2.948)	27.31	126	2.6 ± 0.4	9.54 (3)	0.023	0 (60)
	*S*. *frugiperda*	Dalby	380	0.674 (0.581, 0.781)	9.26	48	2.7 ± 0.2	8.57 (4)	0.073	1.7 (60)
	*S*. *frugiperda*	Nandi	440	1.304 (1.063, 1.612)	119.18	93	1.6 ± 0.1	6.06 (5)	0.300	5.0 (60)
	*S*. *frugiperda*	Wheatvale	376	1.533 (1.268, 1.856)	69.25	109	1.9 ± 0.2	4.14 (4)	0.387	3.3 (60)
	*S*. *frugiperda*	Byee	338	1.451 (1.199, 1.756)	54.96	104	2.0 ± 0.2	4.28 (4)	0.369	7.5 (40)
	*S*. *frugiperda*	Kununurra	480	1.849 (1.469, 2.355)	418.07	132	1.3 ± 0.1	10.98 (6)	0.089	6.7 (60)
	*S*. *frugiperda*	Pooled	4040	1.039 (0.975, 1.107)	84.43	74	1.6 ± 0.1	7.38 (5)	0.194	6.9 (540)
	*H*. *armigera*	New GR	505	0.014 (0.012, 0.016)	0.52	-	2.0 ± 0.2	4.92 (4)	0.296	95.0 (60)
Gamma cyhalothrin	*S*. *frugiperda*	Walkamin	926	0.279 (0.202, 0.403)	14.93	56	1.8 ± 0.2	12.47 (4)	0.014	14.3 (140)
	*S*. *frugiperda*	Mackay	440	0.288 (0.171, 0.450)	30.56	58	1.5 ± 0.2	12.33 (5)	0.030	6.7 (60)
	*S*. *frugiperda*	Bowen	380	0.303 (0.256, 0.358)	7.54	61	2.2 ± 0.2	2.04 (4)	0.728	6.7 (60)
	*S*. *frugiperda*	Ayr	359	0.993 (0.832, 1.184)	25.66	199	2.2 ± 0.2	4.10 (3)	0.251	0 (60)
	*S*. *frugiperda*	Dalby	380	0.406 (0.347, 0.477)	7.88	81	2.4 ± 0.2	2.06 (4)	0.725	1.7 (60)
	*S*. *frugiperda*	Nandi	265	0.760 (0.626, 0.922)	19.80	152	2.2 ± 0.3	5.67 (3)	0.123	-
	*S*. *frugiperda*	Kununurra	379	0.986 (0.818, 1.201)	43.65	197	1.9 ± 0.17	6.06 (4)	0.195	0 (60)
	*S*. *frugiperda*	Pooled	3129	0.433 (0.403, 0.466)	35.20	87	1.6 ± 0.1	4.64 (5)	0.461	7.8 (380)
	*H*. *armigera*	New GR	360	0.005 (0.004, 0.006)	0.11	-	2.9 ± 0.2	3.54 (5)	0.316	100 (60)

† LC_50_ *S*. *frugiperda*/LC_50_ *H*. *armigera*.

### Comparative toxicity in *S*. *frugiperda* and *H*. *armigera*

There was a low level of reduced sensitivity to emamectin benzoate and chlorantraniliprole in *S*. *frugiperda* compared with *H*. *armigera*. Sensitivity to emamectin benzoate was reduced by 3-fold (Kununurra strain) and sensitivity to chlorantraniliprole was reduced by up to 5-fold (Ayr strain). There was 100% mortality in all populations of *S*. *frugiperda* at the diagnostic concentrations of chlorantraniliprole (1.0μg/ml) and emamectin benzoate (0.19μg/ml) known to kill 99.9% of *H*. *armigera* (Table 3).

A high degree of reduced sensitivity to indoxacarb was found in populations of *S*. *frugiperda* compared with a laboratory strain of *H*. *armigera*. The Walkamin strain was 11-fold less sensitive than *H*. *armigera* while the two most tolerant strains were from Kununurra and Ayr which were 61 and 63-fold more tolerant than *H*. *armigera*, respectively. Reduced sensitivity in *S*. *frugiperda* was also evident from survival at the diagnostic dose (12μg/ml) known to kill 99.9% of susceptible *H*. *armigera*. At this dose, survival of *S*. *frugiperda* ranged from 78.0% in the Ayr population to 98.7% in the Byee population (Table 3).

There was also a low level of reduced sensitivity to methoxyfenozide in *S*. *frugiperda* ranging from 3-fold (Dalby strain) to 7-fold (Bowen stain) compared with *H*. *armigera*. There were similar levels of sensitivity to spinosyns in both *S*. *frugiperda* and *H*. *armigera* indicated by toxicity ratios that approximate 1 in most cases. However, spinetoram was significantly more toxic in both species compared with spinosad (Table 4).

There was a small but significant decrease in sensitivity to methomyl *S*. *frugiperda* compared with *H*. *armigera* (average lethal dose ratio 4.5, CI; 3.8162, 5.2757) (Table 5). Methomyl was between 3- and 11-fold more toxic on the laboratory strain of *H*. *armigera* compared with *S*. *frugiperda* while chlorpyrifos was significantly more toxic on *H*. *armigera* than *S*. *frugiperda* (average lethal dose ratio 3.2, CI; 0.806, 1.740) with a lower range of intra-specific variability (2 to 4-fold). Significant levels of survival were observed in *S*. *frugiperda* at the diagnostic concentrations of Group 1 insecticides used to discriminate between resistant and susceptible *H*. *armigera*. Mortality in *S*. *frugiperda* ranged from 31.7% to 63.3% and from 53.3% to 80% for methomyl and chlorpyrifos, respectively (Table 5).

There was clear evidence of reduced sensitivity to synthetic pyrethroids in *S*. *frugiperda* compared with *H*. *armigera*. Gamma cyhalothrin was 56 to 199-fold less toxic on S. *frugiperda* than on *H*. *armigera* while alpha cypermethrin was 44 to 132-fold less toxic on *S*. *frugiperda* than on *H*. *armigera* (Table 5). There were also low levels of mortality at the *H*. *armigera* diagnostic dose, most notably in the Ayr population where 100% of larvae survived at the diagnostic concentrations of both pyrethroids.

### Establishment of diagnostic concentrations of insecticide

The theoretical LC_99.9_ values for emamectin benzoate exceeded the *H*. *armigera* diagnostic concentration (0.19μg/μl of diet) in five of the 11 populations tested. However, empirical testing showed 100% mortality at this dose in all *S*. *frugiperda* populations (Table 3) suggesting that this may be a practicable and reliable method for discriminating between resistant and susceptible genotypes of *S*. *frugiperda*.

The selection of the chlorantraniliprole diagnostic concentration is also supported by the theoretical LC_99.9_ values in the majority of cases with the exception of three populations, Mackay, Chinchilla and Mount Tarampa, where the LC_99.9_ value exceeded 1.0μg/ml. However, empirical mortality observed at this dose produced 100% mortality in all *S*. *frugiperda* strains (Table 3), suggesting this concentration could be suitable and practice test for discriminating between resistant and susceptible genotypes.

The theoretical LC_99.9_ values for indoxacarb ranged from 64.3 to 133.5μg/ml in *S*. *frugiperda* and the diagnostic dose for *H*. *armigera* produced between 78.0% and 98.7% mortality in the 11 strains of *S*. *frugiperda* tested (Table 3). Due to the significant reduction in sensitivity to this insecticide in *S*. *frugiperda* it will be necessary to increase the diagnostic concentration of indoxacarb to a minimum dose of 48μg/ml, compared with 12μg/ml of diet for *H*. *armigera*. This is supported by the empirical mortality of 100% at this higher dose in all *S*. *frugiperda* populations tested.

Diagnostic concentrations of spinosyns have not previously been established for *H*. *armigera*. In the case of spinetoram we found that the LC_99.9_ exceeded the highest concentration tested on *S*. *frugiperda* in only one instance (Wheatvale strain, 1.66μg/ml) and that a dose of 0.75μg/ml produced 100% mortality in all strains tested (Table 4). For spinosad the highest LC_99.9_ value of 6.03μg/ml (Wheatvale strain) also produced 100% mortality in all other strains of *S*. *frugiperda* tested (Table 4). Based on these results and the finding of a very similar dose response in *H*. *armigera* we propose diagnostic concentrations of 0.75 and 6.0μg/ml of diet for spinetoram and spinosad, respectively, for *S*. *frugiperda* and *H*. *armigera*.

For methoxyfenozide a diagnostic concentration of 12.0μg/ml may be considered as a suitable diagnostic concentration for *S*. *frugiperda* based on 100% mortality at this dose in all strain of *S*. *frugiperda* tested (Table 4). However, due to the differential toxicity of methoxyfenozide on the two species, this concentration may not be appropriate for *H*. *armigera* and further calibration on geographically diverse field populations of *H*. *armigera* is required before a diagnostic dose of methoxyfenozide can be recommended for this species.

### Synergism bioassays

The LC_50_ of gamma cyhalothrin was reduced in the Walkamin strain of *S*. *frugiperda* from 0.279μg/μl to 0.004μg/μl by the addition of PBO and resulted in a synergistic ratio of 69.8-fold. Resistance was suppressed to a lesser extent with the addition of PBO to alpha cypermethrin. The LC_50_ for this insecticide was reduced from 0.676 μg/μl to 0.019μg/μl resulting in a synergistic ratio of 35.6 (Table 6). Treatment with a combination of pyrethroid and TPP or DEM did not suppress resistance to either gamma cyhalothrin or alpha cypermethrin (Table 6).

**Table 6:** Toxicity of synthetic pyrethroids with and without synergists on *S*. *frugiperda* determined from topical bioassays using technical grade insecticide.

Insecticide	*n*	LD_50_ [μg/μl] (95% FL)	Fit of probit line			Synergism Ratio^¥^
			Slope ± SE	Χ^2^ (df)	*P*	
Gamma cyhalothrin alone	926	0.279 (0.202, 0.403)	1.8 ± 0.2	12.47 (4)	0.014	-
Gamma cyhalothrin + PBO	1093	0.004 (0.003, 0.006)	1.3 ± 0.2	12.42 (4)	0.015	69.8
Gamma cyhalothrin + DEM	419	0.501 (0.405, 0.610)	1.7 ± 0.2	2.89 (4)	0.576	0.6
Gamma cyhalothrin + TPP	418	0.604 (0.510, 0.714)	2.2 ± 0.2	9.47 (4)	0.050	0.5
Alpha cypermethrin alone	886	0.676 (0.569, 0.793)	1.3 ± 0.1	2.45 (5)	0.784	-
Alpha cypermethrin + PBO	639	0.019 (0.017, 0.022)	2.0 ± 0.1	6.75 (5)	0.240	35.6
Alpha cypermethrin + DEM	420	1.400 (1.136, 1.778)	1.7 ± 0.2	7.07 (4)	0.132	0.5
Alpha cypermethrin + TPP	420	0.978 (0.812, 1.190)	1.9 ± 0.2	1.70 (4)	0.791	0.7

¥ LC_50_ of the strain tested with pyrethroid alone/LC_50_ of the strain tested with pyrethroid and synergist treatment.

## Discussion

A lack of rigour in evaluating naturally occurring baseline responses to insecticides creates a high risk of misleading claims of resistance. False assumptions about resistance can be especially problematic for a newly invasive and potentially resistant insect species, where the development of an insecticide resistance management strategy is necessary. Therefore, evaluation of insecticide toxicity in pest species should take into consideration the range of naturally occurring tolerance present in geographically diverse field populations [42].

The present study documents the intra-specific variation associated with baseline response to insecticides registered under permit for control of *S*. *frugiperda* in Australia [43] and compared these responses to *H*. *armigera*. These comparisons could add predictive value to management decisions for controlling *S*. *frugiperda*, especially when benchmarked against the known practical significance of insecticide resistance in *H*. *armigera*. Baseline data were used to establish concentrations of insecticide that could be implemented in resistance surveillance programs as a first step toward resistance management in *S*. *frugiperda* in Australia.

Spinosyns insecticides spinetoram and spinosad are biologically active compounds derived from fermentation products of the soil Actinomycete *Saccharopolyspora spinosa*. They activate nicotinic acetylcholine receptors (nAChR) and interfere with GABA-gated ion channels of insect nervous systems causing disruption to neuronal activity in insects [44,45]. Australian populations of *S*. *frugiperda* had similar levels of sensitivity to spinetoram compared with a laboratory susceptible strain of *H*. *armigera*. A very low range of variability was found in the response of 11 field strains of *S*. *frugiperda* to this insecticide. These results were consistent with a narrow range of intra-specific variation in spinetoram toxicity in eight strains of *S*. *frugiperda* from China [46].

High susceptibility and low variability in response to spinosad was also found in *S*. *frugiperda* indicating that both spinosyn insecticides are likely to be effective control options on Australian populations. However, resistance to spinosyns has been documented in field and laboratory populations of *S*. *frugiperda* from central and south America [47–49] indicating a capacity for selection of resistance to occur if use of these insecticides increases. As a pre-emptive step in resistance management of spinosyns, the recommended diagnostic doses for spinetoram and spinosad in *S*. *frugiperda* are 0.75 and 6μg/μl of diet, respectively. However, while products containing these active ingredients may be effective, they are more expensive than some of the other options available and so are likely less likely to be used in broadacre systems on commodities with narrow profit margins.

Both emamectin benzoate and chlorantraniliprole are important selective options for management of *H*. *armigera* in Australian broadacre and horticultural farming systems. Emamectin benzoate is derived from naturally occurring macrocyclic lactones isolated from fermentation products of the soil micro-organism *Steptomyces avermitilis*. It binds to GABA-gated chloride channels and causes irreversible activation of channels resulting in disruption of nerve function in lepidopteran species [50]. Chlorantraniliprole binds to RyR receptors in muscle cells of insects causing calcium to be released from sarcoplasmic reticulum. This results in impaired regulation of muscle contraction leading to feeding cessation [51]. While resistance to these insecticides is rare in Australian populations of *H*. *armigera* [37] the use of emamectin benzoate and chlorantraniliprole has the potential to become problematic in crops where the two species occur together as the additional use will increase resistance risk in both species.

Larvae of *S*. *frugiperda* were 2 to 3-fold less sensitive to emamectin benzoate and 2 to 5-fold less sensitive to chlorantraniliprole than larvae of *H*. *armigera*. However, no larvae survived at the diagnostic concentrations established for use in resistance surveillance of *H*. *armigera* [37]. This may indicate that genes that confer practical resistance to these insecticides is not present in Australian populations of *S*. *frugiperda*. This also supports our hypothesis that small differences in intra-specific tolerance to these insecticides is likely to reflect naturally occurring variability inherent in this species. However, a narrow range of susceptibility may not preclude the potential to respond to selection pressure [52]. Moreover, metabolic detoxification is also associated with insect resistance to diamides in the closely related species *Spodoptera litura* (Fabricius) [53]) and it will be important to monitor resistance as a key component of resistance management for *S*. *frugiperda*.

Although of diamide insecticides are highly effective in *S*. *frugiperda* [54–56] frequent use has resulted in increased frequency of chlorantraniliprole resistance in *S*. *frugiperda* in Brazil [57] and Puerto Rico [48]. Bolzan et al. (2019) [57] also showed 15% survival of larvae heterozygous for resistance when treated with a field rate of chlorantraniliprole, indicating a high risk of practical resistance evolution to diamide insecticides in *S*. *frugiperda*. However, the putative target site for diamide resistance (the ryanodine receptor) was not identified in 34 geographically diverse populations from the Americas, Africa and Indonesia [58]. Therefore, proactive steps in resistance management are recommended to monitor changes in *S*. *frugiperda* sensitivity to these key insecticides. We have determined that concentrations of 0.19μg/μl of diet for emamectin benzoate and 1.0μg/μl of diet for chlorantraniliprole could be used in resistance surveillance programs to effectively discriminate between resistance and susceptible phenotypes of *S*. *frugiperda*.

Methoxyfenozide is an insect growth regulator which mimics the moulting hormone and elicits toxicity by causing premature and lethal moult by direct stimulation of ecdysteroid receptors [59]. The highly specific mode of action and favourable non-target and environmental profile of methoxyfenozide is highly compatible with IPM systems [60,61]. Although methoxyfenozide had lower toxicity that the spinosyns, emamectin benzoate and chlorantraniliprole, there was narrow variation between populations of *S*. *frugiperda* suggesting this insecticide may be useful for achieving population suppression in low pressure situations and for providing an additional rotation option for resistance management.

Indoxacarb blocks voltage-dependent sodium channels, preventing influx of sodium into neurons [62]. This insecticide had lower toxicity on *S*. *frugiperda* than *H*. *armigera* and sensitivity was highly variable between *S*. *frugiperda* populations. The use of indoxacarb on a global scale may not explain an increase in selection for resistance to this insecticide and it is possible that selection of generalist mechanisms of resistance such as systems of metabolic detoxification may also confer reduced sensitivity to indoxacarb. Indoxacarb toxicity was found to be relatively lower than other active constituents tested on *S*. *frugiperda* populations from China [46] and India [56].

The Group 1 insecticides (organophosphates and carbamates) target the acetylcholinesterase enzyme (AChE) and resistance can be associated with mutations in the *ace-1* gene as a result of amino acid substitutions at active site of the enzyme [63]. Sensitivity to Group I insecticides was reduced in *S*. *frugiperda* compared to laboratory susceptible *H*. *armigera*. Average larval survival of *S*. *frugiperda* exposed to the diagnostic concentration used to discriminate between methomyl-resistant and -susceptible *H*. *armigera* was 52%. This level of reduced sensitivity to methomyl is consistent with the detection of mutations at the AChE in samples from various locations throughout northern Qld and northern WA. All *S*. *frugiperda* larvae tested from these locations had at least one mutation while 68 per cent was found to be homozygous for this mutation, suggesting that applications of organophosphates and carbamates would not provide effective control of *S*. *frugiperda* [64]. Recently, amino acid substitutions associated with Group 1 resistance were also found to occur at high frequency in populations of *S*. *frugiperda* from China [46], Africa, Indonesia, Puerto Rica and Brazil [58].

Australian populations of *S*. *frugiperda* were highly insensitive to pyrethroids with very high levels of survival at the diagnostic dose of both alpha cypermethrin and gamma cyhalothrin, indicating these insecticides are highly unlikely to be effective in controlling *S*. *frugiperda*. Previous studies demonstrated that detoxification by microsomal oxidases, glutathione S-transferases and carboxylesterases are causal for resistance to pyrethroids in *S*. *frugiperda* [23,24]. McComic et al. (2020) [65] did not detect target site resistance mediated by the VGSC in a *S*. *frugiperda* population with 767-fold resistance to gamma cyhalothrin. A recent study conducted on 34 geographically distinct populations of *S*. *frugiperda* found a very low frequency (1.8%) of heterozygous resistance of one mutation (L1014F) at the VGSC in Indonesian *S*. *frugiperda* while there were no mutations at the VGSC in populations from Brazil, Puerto Rico or Kenya [58]. In contrast, pyrethroid resistant strains of *S*. *frugiperda* from Brazil carried mutations that resulted in three amino acid substitutions at the VGSC (T929I, L932F and L1014F) which are known to confer target site resistance to pyrethroids in several arthropod species [23].

Results from the present study suggest that PBO-suppressible metabolic processes such as enhanced detoxification by cytochrome P450 monooxygenases are likely to be important mechanisms conferring resistance to pyrethroids, while glutathione-S-transferase and carboxylesterase don’t appear to play major roles in resistance in Australian *S*. *frugiperda*. Similarly, in synergist studies conducted in populations of *S*. *frugiperda* from China, P450 monooxygenases were found to suppress pyrethroid resistance to greater extent than glutathione-S-transferase and carboxylesterase (Zhao et al. 2020). Whole-genome sequencing also confirmed increased copy numbers of P450 genes from invasive populations of *S*. *frugiperda* sampled from 12 geographic populations in North and South America, Africa, India and China compared with native populations from these regions [66]. Increased copy number of P450 genes was also correlated with increased resistance to deltamethrin in a strain of *S*. *frugiperda* from Puerto Rico [25], while a high level of expression of several P450s was observed in a field strain of *S*. *frugiperda* from Brazil with resistance to several insecticidal classes [26].

The data presented herein suggests there may be early signs of differential resistance in some intensive horticultural regions of northern Australia such as Ayr and Kununurra which may indicate a heightened risk of selection for resistance alleles if insecticide use to target *S*. *frugiperda* populations in these areas increases. There could be several reasons for the inter- and intra-specific variation in sensitivity to insecticides observed in the present study including the expression of insecticide-induced hormesis which is a dose-response phenomenon characterised by a stimulatory effect associated with exposure to low or sublethal doses of toxic compounds that are lethal at higher doses [67,68], and/or induction of detoxification enzymes by up-regulation/overexpression. Both scenarios can lead to increased capacity of arthropods to detoxify insecticidal compounds and may contribute to decreased pest sensitivity to insecticides resulting in increased potential for field control failures [69,70]. Hormesis effects from low dose exposure to insecticides has been demonstrated in noctuid pests [71,72] including *S*. *frugiperda* [73]. Therefore, careful management of selective insecticidal options is recommended to reduce both lethal and sub-lethal exposure to any one mode of action group.

In Australia there is a well-established *H*. *armigera* resistance surveillance program which supports a highly successful insecticide resistance management strategy for this pest which is widely adopted by industries [27,29]. There is now an urgent need for a similar surveillance program to support strategic resistance management in *S*. *frugiperda* to not only mitigate resistance risk in this species but to avoid undermining resistance management in *H*. *armigera*. These strategies should rely on principles designed to minimise development of insecticide resistance firstly to minimise the use of insecticides and reduce selection pressure by optimising insecticide applications [74]. The most effective way to maximise chemical control of *S*. *frugiperda* is to conduct regular surveillance to alert growers of outbreaks and provide the opportunity for early intervention before larvae establish in whorls of plants, at which point optimal control will be more difficult to achieve. The second component of resistance management should incorporate tactics that will avoid selection of resistance mechanisms. This will be most effectively achieved by chemical rotation of products with different modes of action (without cross-resistance) alternated between product use ‘windows’ while taking into account the length of a pest generation to ensure that consecutive generations are not exposed to the same mode of action group [75,76].

Any strategy for managing resistance should be designed with integrated control in mind and incorporate as many different mitigation tactics as possible including the use of synthetic and biological insecticides, beneficial arthropods, cultural practices, crop rotation, host-plant resistance and chemical attractants/deterrents [75]. Any one or more of these tactics may reduce the number of insecticide applications, thus lowering selection pressure on pest populations.

Our study demonstrates that there is high susceptibility to selective insecticides in Australia. Timely applications of these products on above threshold populations is recommended for optimising applications. However, the use of these products is not without risk to non-target species and the use of chlorantraniliprole and spinosad in particular has significant negative impacts on generalist predators and parasitic wasps [18–20]. The use of registered biological options including foliar Bt insecticides and the *S*. *frugiperda* multiple nucleopolyhedrovirus (SfMNPV) insecticide is highly recommended to maximise density and preserve diversity of beneficial arthropods which have the potential to contribute to suppression of *S*. *frugiperda* populations [20], while spreading resistance risk across a wider range of mode of action groups.

## Conclusion

An evidence-based assessment of resistance status in newly established pest species is important for applying suitable approaches in pest management. Since the arrival of *S*. *frugiperda* in Australia in 2020 the risk of spray failures and other consequences of insecticide resistance in this pest are of serious concern to Australian agricultural industries. Diagnostic tests established in this study will be useful tools to increase capacity for early detection and future monitoring of resistance in this now widely dispersed population of *S*. *frugiperda* in Australia. Our results provide foundational information for optimising the control of *S*. *frugiperda* while maximising natural enemy populations, the benefits of which will be destroyed by broad-spectrum insecticides that are unlikely to provide effective control in this species. The future implementation of integrated control and resistance management strategies will be critically important for ensuring that pivotal insecticides remain sustainable for management of *S*. *frugiperda* in Australian farming systems.
